# Prostate cancer: what is the right message?

**DOI:** 10.1590/S1679-45082015ED3595

**Published:** 2015

**Authors:** Sidney Glina, Jacyr Pasternak

**Affiliations:** 1 Hospital Israelita Albert Einstein, São Paulo, SP, Brazil.

The general public and the non-specialist physicians watch a debate that, at least, leaves some doubts regarding management.

Several organizations have criticized the use of the prostate specific antigen (PSA) for early diagnosis of prostate cancer, based on the recommendation against this screening by the United States Preventive Services Task Force (USPSTF).^(1)^ This statement was based on a study from the Unites States, which did not demonstrate reduced survival of prostate cancer patients, or that the use of PSA significantly increased the diagnosis of this tumor.^(2)^ Moreover, many prostate biopsies are unnecessary considering that the PSA has low specificity. The diagnosis of indolent tumors that do not progress made PSA a major villain. This document, published in 2012, has already led to a decreased number of tests ordered by American clinicians.^(3)^

On the other hand, several urology societies still recommend men aged between 55 and 69 years to be submitted to the test, provided all risks are explained, since the “benefits surpass the risks”.^(4)^ In Brazil, the “Blue November” campaign, supported by the Brazilian Society of Urology (SBU), is launched every year, in November, when males are informed about health care, specially prostate cancer.^(5)^

Putting the emotional debate aside, several aspects should be addressed. The study that was the basis for the USPSTF recommendation has been criticized by contamination of the control group (males who should not have their PSA dosed and many of them ordered the test at their own discretion). Yet, an European study^(6)^ with a similar design showed that early diagnosis of prostate cancer decreased the risk of death by 21%, in 11 years. It is true that a meta-analysis including these two investigations demonstrated that early diagnosis of prostate cancer did not result in lower mortality rates.^(7)^

The current level of knowledge does not allow us to simply state early diagnosis of prostate cancer should not be searched and give up this condition. Prostate cancer is the most frequent neoplasm in males and ranks second in mortality, after lung cancer.

Giving up the early diagnosis policies will take us back to 1987 – the pre-PSA era - when patients searched medical care already presenting metastases and with no possibility of curative treatment. Unarguably, the use of PSA radically decreased the number of cases with advanced and metastatic tumors, unlike mammography, which did not reduced the cases of breast cancer.^(8)^

Prostate cancer screening is far from being perfect, but can be and has been improved. The use of other indicators, such as free/total PSA ratio and multiparametric resonance magnetic, in addition to search for new markers, have decreased the number of unnecessary biopsies and helped detecting the cases of indolent tumors.

In 1980, the estimated life expectancy for males in Brazil was 59.6 years.^(9)^ It is known that untreated high-risk localized prostate tumor leads to death within 10 years.^(10)^ Therefore, not making its diagnosis in men aged 50 years would not change much the average survival of Brazilian individuals, at that time. In 2007, the life expectancy was 68.7 years,^(9)^ and this impact on survival may be significant, and even more, in the developed countries, where life expectancy is higher.

Many questions have not been answered yet, regarding the economic impact of treating a larger number of metastic cases; the impact of PSA and early diagnosis in prolonging survival of prostate cancer patients; and the impact of giving up screening of males aged 45 to 50 years, considering that high-risk tumors occur quite often.

Before having all these answers, it seems irresponsible to not inform patients about our doubts and simply state that an early diagnosis should not be made. The decision must be shared and, ultimately, the patient will choose what to do – but aware of the risks of his option.
