# Supplementary material for: Prostate cancer: what is the right message?
Source: Einstein (Sao Paulo). 2015 Oct-Dec;13(4):vii–viii. doi: 10.1590/S1679-45082015ED3595 (PMC4878648; doi:10.1590/S1679-45082015ED3595)
Supplement: Translation

# Translation: Câncer de próstata: qual a mensagem correta?

**Authors:** Sidney Glina, Jacyr Pasternak

**Affiliations:** 1 Hospital Israelita Albert Einstein, São Paulo, SP, Brasil.

O público em geral e os médicos não especialistas assistem a um debate que, no mínimo, deixa dúvidas sobre como se comportar.

Várias organizações vêm condenando o uso do antígeno prostático específico (PSA) para o diagnóstico precoce do câncer de próstata, baseadas na recomendação da *United States Preventive Services Task Force* (USPSTF)*,* ^(1)^ que condenou o rastreamento dessa neoplasia. Esta declaração baseou-se em um estudo americano, que não mostrou diminuição da sobrevida dos pacientes com câncer de próstata e nem que o uso do PSA aumentou significativamente o diagnóstico desse tumor.^(2)^ Associado ao número de biópsias prostáticas desnecessárias, pois o PSA tem baixa especificidade, o diagnóstico de tumores indolentes, que não evoluiriam, transformaram o PSA em um grande vilão. O documento de 2012 já produziu uma redução no número de exames solicitados pelos clínicos americanos.^(3)^

Por outro lado, várias sociedades de urologia ainda recomendam que homens entre 55 e 69 anos façam os exames, desde que esclarecidos de todos os riscos, pois os “benefícios ultrapassariam os riscos”.^(4)^ No Brasil, o “Novembro Azul” é uma campanha apoiada pela Sociedade Brasileira de Urologia (SBU), realizada anualmente no mês de novembro, na qual o homem é esclarecido sobre os cuidados com sua saúde, especialmente sobre o câncer da próstata.^(5)^

Deixando de lado o debate emocional, vários aspectos precisam ser considerados. O estudo que serviu de base para a recomendação da USPSTF vem sendo criticado pela contaminação do grupo controle (homens que não deveriam fazer dosagens de PSA e boa parte o fez por conta própria). Por outro lado, estudo europeu^(6)^ que tinha desenho semelhante mostrou que o diagnóstico precoce do câncer de próstata diminuiu o risco de morte em 21% em 11 anos. Verdade que uma metanálise, que incluiu os dois estudos, mostrou que o diagnóstico precoce do câncer de próstata não reduziu a mortalidade.^(7)^

O estágio atual do conhecimento não pode simplesmente dizer que não deve se buscar o diagnóstico precoce do câncer de próstata e abandonar esta doença. O câncer de próstata é a neoplasia que mais afeta os homens e é o segundo que mais mata, após o câncer de pulmão.

O abandono da políticas de diagnóstico precoce vai nos devolver a 1987, na era pré-PSA, quando os pacientes buscavam ajuda médica com quadros já metastáticos e sem possibilidade de tratamento curativo. É inquestionável que o uso do PSA diminuiu radicalmente o número de casos com tumores avançados e metastáticos, ao contrário da mamografia, que não diminuiu estes casos na neoplasia de mama.^(8)^

O rastreamento do câncer de próstata está longe de ser perfeito, mas pode e está sendo aperfeiçoado. O uso de outros indicadores, como a relação do PSA livre/total, a ressonância magnética multiparamétrica e a busca de novos marcadores tem diminuído o número de biópsias desnecessárias e ajudado a identificar os casos de tumores indolentes.

Em 1980, a expectativa de vida média do homem no Brasil era de 59,6 anos,^(9)^ e, como sabemos que o tumor de próstata de alto risco localizado não tratado leva ao óbito em 10 anos,^(10)^ provavelmente não diagnosticá-lo aos 50 anos não alterava muito a sobrevida média do homem brasileiro. Em 2007, esta expectativa média passou a ser de 68,7 anos,^(9)^ e esse impacto na diminuição da sobrevida pode ser significativo – e maior ainda nos países desenvolvidos onde a expectativa é maior.

Muitas questões ainda precisam ser respondidas, entre elas qual vai ser o impacto econômico de se tratar um número maior de casos metastáticos; qual o impacto do PSA e do diagnóstico precoce no prolongamento da sobrevida dos pacientes com câncer de próstata; e qual o impacto do abandono do rastreamento em homens entre 45 e 50 anos, quando muitas vezes os tumores são de alto risco.

Antes de se ter todas as respostas, parece irresponsável não informar aos pacientes todas as nossas dúvidas e dizer simplesmente que não se deve fazer o diagnóstico precoce. A decisão deve ser compartilhada e, em última análise, o paciente escolhe o que fazer – mas consciente dos riscos de sua escolha.
